# Supplementary material for: High affinity binding of SARS-CoV-2 spike protein enhances ACE2 carboxypeptidase activity
Source: bioRxiv. 2020 Jul 1:2020.07.01.182659. Preprint. [Version 1] doi: 10.1101/2020.07.01.182659 (PMC7337377; doi:10.1101/2020.07.01.182659)

bioRxiv preprint doi: https://doi.org/10.1101/2020.07.01.182659. this version posted July 1, 2020. The copyright holder for this preprint (which was not certified by peer review) is the author/funder. This article is a US Government work. It is not subject to copyright under 17 USC 105 and is also made available for use under a CC0 license.

Supplementary figure 1 SARS-CoV-2 spike protein enhanced ACE2 cleavage of fluorogenic caspase-1 substrate and bradykinin analog in a concentration dependent manner with increased concentration of NaCl in the assay buffer. A) and C) Kinetic reading of relative fluorescence units (RFU) monitoring the hydrolysis of Mca-YVADAPK-Dnp(20uM) and Mca-RPPGFSAFK-Dnp (20uM) in the presence of SARS-CoV-2 spike protein at indicated final concentrations in the enzymatic assays with 0.3M NaCl. The maximum reading is limited to 100000 RFU on Synergy H1 plate reader. B) and D) Kinetic reading of relative fluorescence units (RFU) monitoring the hydrolysis of Mca-YVADAPK-Dnp(20uM) and Mca-RPPGFSAFK-Dnp (20uM) in the presence of SARS-CoV-2 spike protein at indicated final concentrations in the enzymatic assays with 1.0M NaCl.

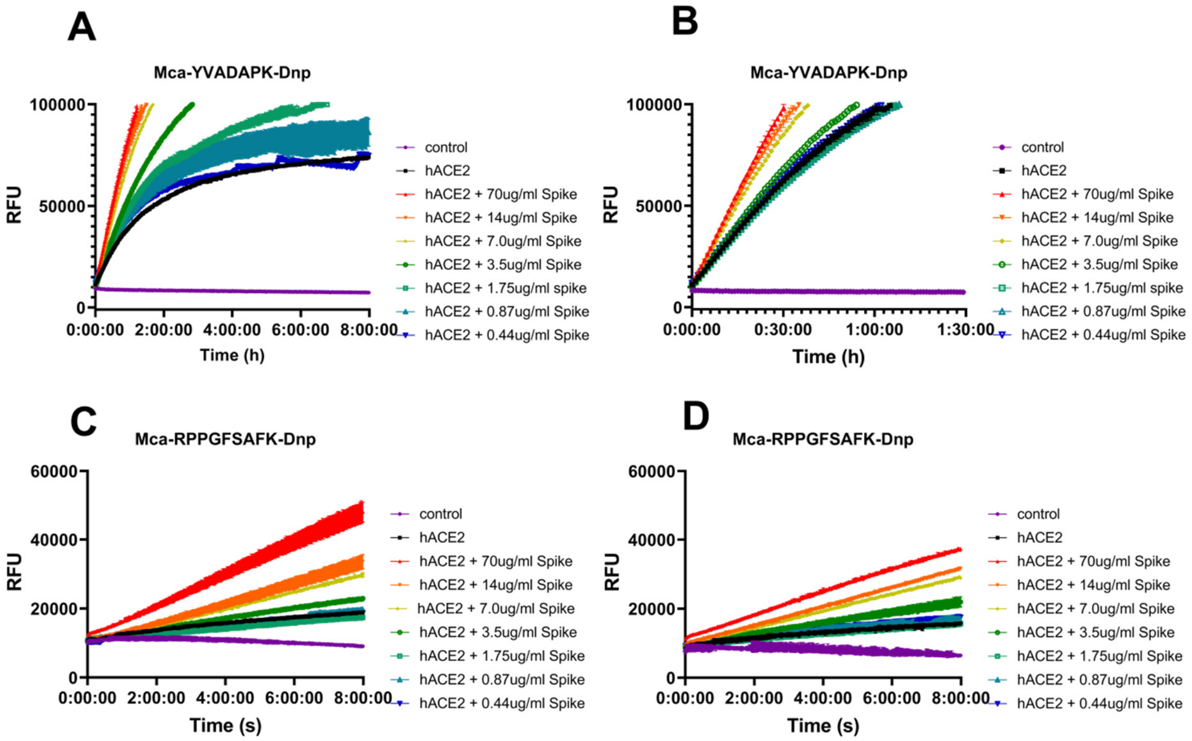

bioRxiv preprint doi: https://doi.org/10.1101/2020.07.01.182659. this version posted July 1, 2020. The copyright holder for this preprint (which was not certified by peer review) is the author/funder. This article is a US Government work. It is not subject to copyright under 17 USC 105 and is also made available for use under a CC0 license.

Supplementary figure 2 Calibration of Synergy H1 plate reader with fluorescent Mca-Pro-Leu-OH peptide to obtain relative fluorescent unit to molarity conversion factor. All readings were carried out in 110 ul of enzymatic assay buffers with various concentrations of Mca-Pro-Leu-OH peptide.

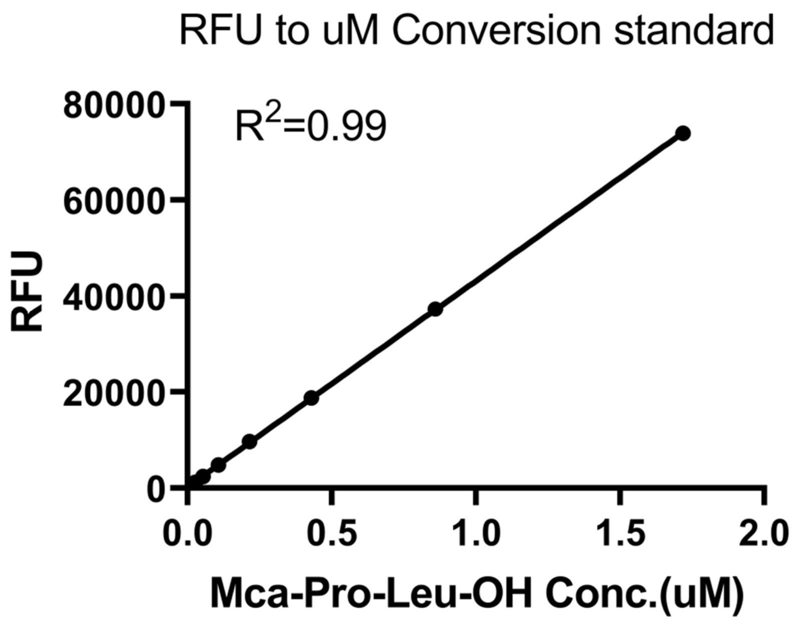

bioRxiv preprint doi: https://doi.org/10.1101/2020.07.01.182659. this version posted July 1, 2020. The copyright holder for this preprint (which was not certified by peer review) is the author/funder. This article is a US Government work. It is not subject to copyright under 17 USC 105 and is also made available for use under a CC0 license.

Supplementary figure 3 Competition of Bradykinin(BK), des-Arg9-BK, and Ang II peptide with Mca-RPPGFSAFK-Dnp (10uM) for ACE2 hydrolysis. A),C) and E) panels showing the substrates competition in the presence of SARS-CoV-2 RBD protein at different concentrations. B),D) and F) panels showing the substrates competition in the absence of SARS-CoV-2 RBD protein at different concentrations. G) Comparison of RFU during ACE2 hydrolysis of Mca-RPPGFSAFK-Dnp in the presence of competitive substrate at time 1h. The pairwise p-value statistics were calculated between (hACE2 + SARS-CoV-2 RBD) and corresponding concentrations of competitive peptides. H) Comparison of RFU during ACE2 hydrolysis of Mca- RPPGFSAFK-Dnp in the absence of competitive substrate at time 1h. The pairwise p-value statistics were calculated between hACE2 and corresponding concentrations of competitive peptides.

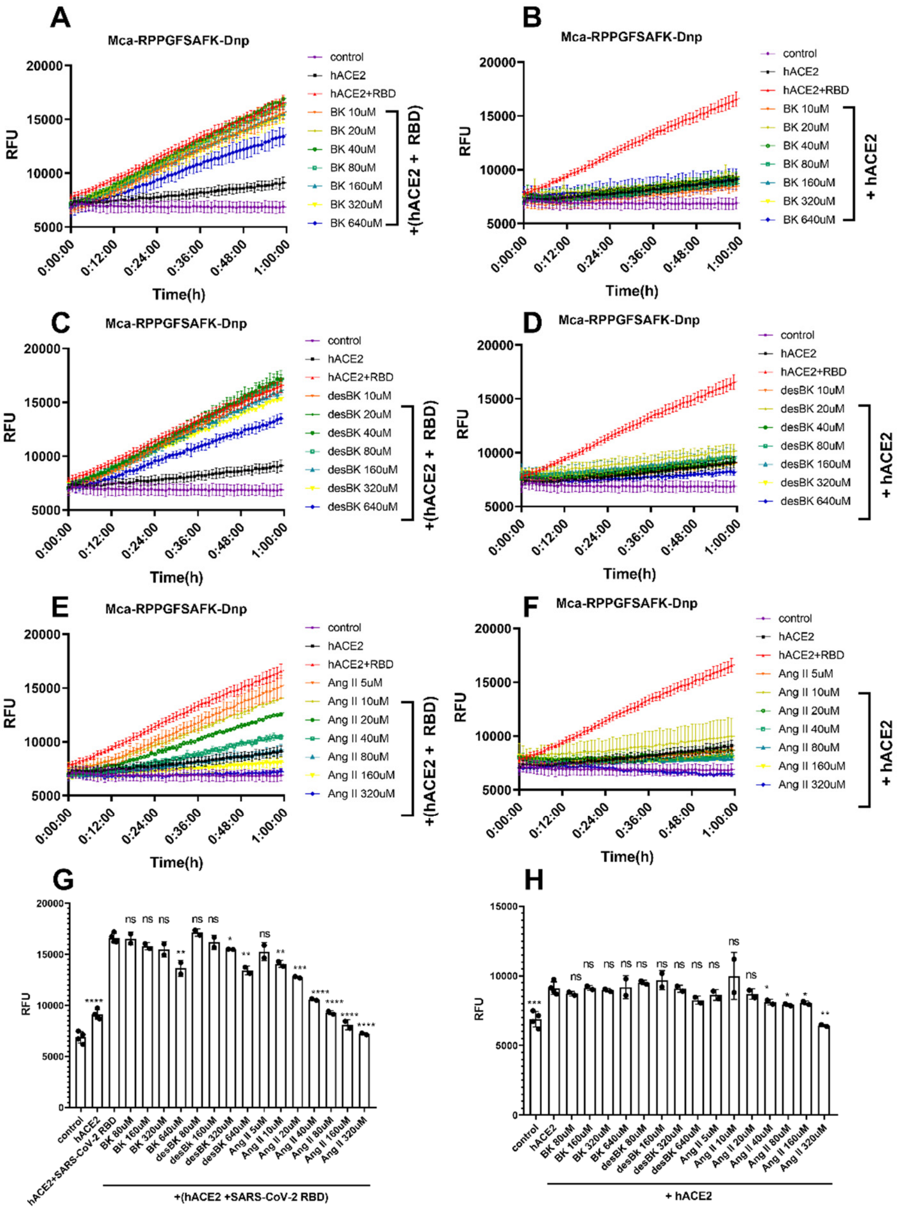

Supplement: 1 [file NIHPP2020.07.01.182659-supplement-1.pdf]
